# Effect of sitagliptin on diabetes-induced hyperpermeability of blood-retinal barrier components

**DOI:** 10.1038/s41433-025-03924-w

**Published:** 2025-07-15

**Authors:** Rafael Simó, Hugo Ramos, Marta García-Ramírez, Cristina Hernández

**Affiliations:** 1 Diabetes and Metabolism Research Unit, Vall d’Hebron Research Institute, Barcelona, Spain; 2 Centro de Investigación Biomédica en Red de Diabetes y Enfermedades Metabólicas Asociadas (CIBERDEM), Instituto de Salud Carlos III (ICSIII), Madrid, Spain; 3 Department of Medicine, Universitat Autònoma de Barcelona, Barcelona, Spain

**Keywords:** Mechanisms of disease, Retinal diseases

The important role of neurodegeneration in the pathogenesis of diabetic retinal disease (DRD) and the evidence that several neuroprotective retinal factors are downregulated in diabetes have led to propose replacement treatment via eyedrops of these neuroprotective factors as a new therapeutic strategy [1, 2]. Glucagon-like peptide-1 (GLP-1) is one of them; however, it presents low stability and rapid degradation by dipeptidyl peptidase-4 (DPP-4), which is more abundant in diabetic than in non-diabetic retina [2]. To overcome this issue, the use of topical administration of DPP-4 inhibitors (DPP-4i) has been proposed [2]. These drugs not only reproduce the positive GLP-1 effects but even have other beneficial actions unrelated to GLP-1R activation that should be elucidated.

This study examines for the first time whether sitagliptin (a DPP-4i), in absence of GLP-1, prevents hyperpermeability on the cells forming the outer and inner blood-retinal barrier (BRB) under conditions that simulate the *diabetic milieu*.

For that purpose, ARPE-19 and human retinal endothelial cells (HREC), were cultured and exposed to five experimental conditions for three days: a) Physiological condition (5.5 mmol/L D-glucose), b) High glucose condition (25 mmol/L D-glucose); c) High glucose condition (25 mmol/L D-glucose) with sitagliptin phosphate monohydrate (Y0001812, Merck KGaA) (40 µg/mL); d) *Diabetic milieu*: 25 mmol/L D-glucose + interleukin 1 beta (IL-1β) (10 ng/mL), TNF-α (25 ng/mL) and recombinant human VEGF (25 ng/mL). e) *Diabetic milieu* with sitagliptin (40 µg/mL). Then, paracellular permeability was assessed using the FITC-Dextran method.

Exposure to high glucose, with or without sitagliptin, did not significantly affect dextran permeability (40 kDa) in either cell type. However, when IL-1β, TNF-α and VEGF were added (diabetic condition), the permeability significantly increased (Fig. 1A, B). Notably, this cytokine-induced increase was prevented when ARPE-19 and HREC cells were cultured with sitagliptin (Fig. 1A, B).

**Fig. 1:**
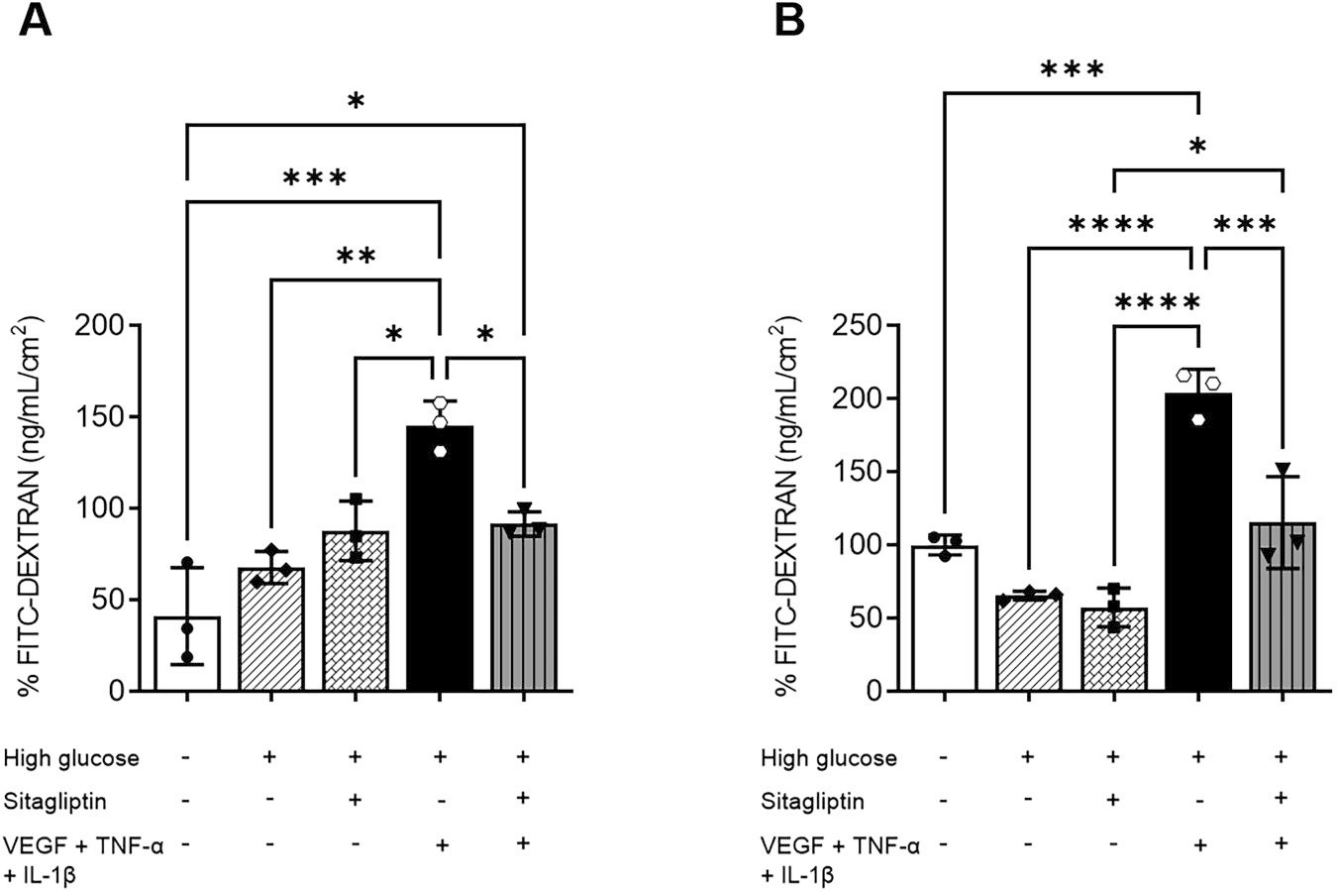
Permeability assays in ARPE-19 and HREC lines using the FITC-Dextran method. Bar graphs illustrating the mean values of Dextran permeability for the different tested conditions in both HREC (**A**) and ARPE-19 (**B**) cell lines. Results are presented as the difference between the concentration of Dextran at the end of the experiment (90 min) less the initial concentration (3 min). Graph bars are displayed as the mean value followed by the standard deviation. White bars: euglycemic condition (5 mM); white bars with diagonal hatching: high glucose condition (25 mM); white bars with a diagonal brick pattern: sitagliptin effect on high glucose conditions; black bars: diabetic milieu (high glucose + VEGF, TNF-α and IL-1β); grey bars with vertical hatching: sitagliptin effect on diabetic milieu. *n* = 3. *p < 0.05, **p < 0.01, ***p < 0.001, ****p < 0.0001.

These results demonstrate that sitagliptin inhibits the hyperpermeability induced by the *diabetic milieu* on both endothelial and RPE cells. This effect cannot be attributed to GLP-1 because HREC and RPE cells do not produce GLP-1.

Previous studies on the effect of sitagliptin on endothelial cells have generated controversial results. The origin of endothelial cells (micro or vascular) is very important in evaluating the previously reported results because gene expression patterns depend on vascular beds [3]. Even at the microvascular bed level, the use of cells from non-human species (i.e. bovine endothelial cells) versus HRECs can significantly impact the experimental outcomes. The selection of the inflammatory mediators (type and doses), as well as VEGF concentration is also crucial. Previous reports have predominantly used TNF-α or VEGF, whereas we have mimicked the diabetic milieu using three of the primary drivers of DRD development in humans simultaneously: hyperglycaemia, inflammation (TNF-α and IL-1β) and VEGF. Therefore, we provide first evidence on the beneficial effects of sitagliptin in such a detrimental environment.

In conclusion, by using a methodology that closely reproduce the conditions that occur in the human retina, our results suggest that sitagliptin is able to prevent diabetes-induced vascular leakage. This action is unrelated to its ability to prevent GLP-1 degradation and point to GLP-1R independent mechanisms as additional players in the beneficial effects of sitagliptin in DRD.

## Data Availability

The data presented in this study are available in the article.
